# Microglia attenuate the kainic acid‐induced death of hippocampal neurons in slice cultures

**DOI:** 10.1002/npr2.12086

**Published:** 2019-12-03

**Authors:** Tasuku Araki, Yuji Ikegaya, Ryuta Koyama

**Affiliations:** ^1^ Laboratory of Chemical Pharmacology Graduate School of Pharmaceutical Sciences The University of Tokyo Tokyo Japan; ^2^ Center for Information and Neural Networks Suita City Japan

**Keywords:** epilepsy, hippocampus, kainite, microglia, neuroprotection

## Abstract

**Background:**

Status epilepticus‐induced hippocampal neuronal death, astrogliosis, and the activation of microglia are common pathological changes in mesial temporal lobe epilepsy (mTLE) with resistance to antiepileptic drugs. Neuronal death in mTLE gradually progresses and is involved in the aggravation of epilepsy and the impairment of hippocampus‐dependent memory. Thus, clarifying the cellular mechanisms by which neurons are protected in mTLE will significantly contribute to the treatment of epilepsy. Here, mainly using hippocampal slice cultures with or without the pharmacological depletion of microglia, we directly examined whether microglia, the resident immune cells of the brain that can act either neurotoxically or in a neuroprotective manner, accelerate or attenuate kainic acid (KA)‐induced neuronal death in vitro.

**Methods:**

Hippocampal slice cultures were treated with KA to induce neuronal death in vitro. Clodronate‐containing liposomes or PLX3397 was used to deplete microglia in hippocampal slice cultures, and the effect on KA‐induced neuronal death was immunohistochemically assessed.

**Results:**

The loss of microglia significantly promoted a decrease in neuronal density in KA‐treated hippocampal slice cultures.

**Conclusion:**

Our results suggest that microglia are neuroprotective against KA‐induced neuronal death in slice cultures.

## Abstract 2

We investigated the role of microglia in kainic acid‐induced neuronal death using hippocampal slice cultures.We found that pharmacological removal of microglia from cultured hippocampal slices enhanced kainic acid‐induced neuronal death. These results suggest that microglia are neuroprotective against kainic acid‐induced neuronal death.

## Graphical abstract

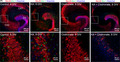

## 1 INTRODUCTION

Mesial temporal lobe epilepsy (mTLE) is one of the most common types of intractable epilepsy in adults, and approximately 30% of patients exhibit resistance to antiepileptic drugs. A common pathological feature of mTLE with drug resistance is characterized by pathological and histological changes such as gliosis and neuronal death in the CA1 and CA3 areas of the hippocampus.^1^ Neuronal death in mTLE gradually progresses and can induce memory impairment and a prognosis in which the hippocampus is extracted for treatment.^2^, ^3^, ^4^ However, the cellular and molecular mechanisms underlying these pathological changes remain largely unknown, and therapeutic strategies to prevent neuronal death have not been established.

It has been reported that microglia are activated in mTLE patients.^5^ Microglia, the immune cells in the central nervous system (CN), scan the brain by continuously and dynamically moving their processes. They maintain brain homeostasis by removing dead cells and cell debris.^6^, and overactivated microglia can cause inflammation by releasing inflammatory cytokines.^7^ Whether microglia play neuroprotective or neurotoxic roles in neurodegenerative diseases varies greatly depending on the type of disease and the progression of the disease.^8^, ^9^ Microglia are also involved in the process of neuronal death in various neurodegenerative diseases, but their role in mTLE has not been clarified.^10^ In this study, cultured hippocampal slices were treated with KA, a prototypic kainate receptor agonist often used to induce status epilepticus in rodents to model mTLE, to model neuronal death in vitro. The role of microglia in neuronal death was assessed by pharmacologically depleting these cells.

## 2 MATERIALS AND METHODS

### 2.1 Animals

In the present study, animal experiments were performed with the approval of the animal experiment ethics committee of the University of Tokyo (approval number: 24‐70) and according to the University of Tokyo's guidelines for the care and use of laboratory animals. C57BL/6J mice (SLC, Shizuoka, Japan) were housed in cages under standard laboratory conditions (a 12‐hours light/dark cycle and free access to food and water). All efforts were made to minimize the animals’ suffering and the number of animals used. To prepare hippocampal slice cultures, mouse pups were decapitated after they were deeply anesthetized on ice.

### 2.2 Hippocampal slice culture

To prepare slice cultures, P6 mouse brains were sectioned into 400‐μm‐thick horizontal slices using a DTK‐1500 vibratome (Dosaka, Kyoto, Japan) in aerated, ice‐cold Gey's balanced salt solution (GBSS) containing 36 mmol/L glucose, as previously described.^11^ Briefly, the entorhinohippocampal regions of the slices were dissected and incubated for 30‐90 minutes at 4°C in cold incubation medium containing minimal essential medium (MEM), 9.0 mmol/L Tris, 22.9 mmol/L HEPES, and 63.1 mmol/L glucose supplemented with penicillin and streptomycin. Following incubation, the slices were placed on Omnipore membrane filters (JHWP02500; Merck Millipore, Billerica, MA, USA) on doughnut plates (Hazai‐Ya, Tokyo, Japan).^12^ in a solution containing 50% MEM, 25% horse serum (26050‐088; HS, heat‐inactivated and filter‐sterilized, Gibco, Grand Island, NY, USA), 25% HBSS, 6.6 mmol/L Tris, 16.9 mmol/L HEPES, and 4.0 mmol/L NaHCO_3_ supplemented with 29.8 mmol/L glucose and 1% gentamicin sulfate solution (16672‐04; Nacalai Tesque, Kyoto, Japan). Finally, the slices were cultured at 35°C in a humidified incubator with 5% CO_2_ and 95% air. The culture medium was changed twice a week.

At 7 days in vitro (DIV), the culture medium was replaced with culture medium containing 20 μmol/L KA (0222; Tocris, Bristol, UK) and treated for 24 hours. After KA treatment, the slices were rinsed carefully three times with PBS, and the KA‐containing medium was changed to fresh medium.

### 2.3 Removal of microglia

Two reagents were used to deplete microglia from slice cultures. First, we used the liposomal clodronate Clophosome‐A (F70101C‐A; FormuMax, Sunnyvale, CA, USA), which is known to effectively deplete macrophages in the mouse spleen after a single intravenous or intraperitoneal administration.^13^, ^14^ At 0 DIV and 3 DIV, Clophosome‐A (0.05 mg/mL) was added to the culture medium for 24 hours. In the control group, the same amount of control anionic liposomes (F70101‐A; FormuMax, Sunnyvale, CA, USA) was added to the medium. After Clophosome‐A treatment, the slices were rinsed carefully three times with warmed PBS and cultured with fresh medium.

Next, we used PLX3397 (also known as pexidartinib, CS‐4256; Monmouth Junction, NJ, USA), a tyrosine kinase inhibitor of colony‐stimulating factor 1 receptor. PLX3397 has been widely used to deplete microglia in rodents.^15^ PLX3397 was dissolved in DMSO (100 mmol/L) and stored at −18℃. From 0 DIV to 7 DIV, PLX3397 (30 mmol/L) was added to the culture medium. The same amount of DMSO was added to the culture medium in the control group. After the PLX3397 treatment, the slices were rinsed carefully three times with warmed PBS and cultured with fresh medium.

### 2.4 Kainic acid injection in vivo

Six‐week‐old mice were deeply anesthetized by the intraperitoneal administration of xylazine (10 mg/kg) and Somnopentyl (27 mg/kg). Next, the mice were subcutaneously administered lidocaine hydrochloride (1.5 mg; 0.1 mL) for local anesthesia. Then, the scalp was cut to expose the skull, and holes were carefully made through the skull with a hand‐held drill at AP = 2.0 mm and ML = +1.5 mm from bregma. Finally, 50 nL of KA (20 mmol/L in saline) was administered at DP = +2.0 mm from the skull surface with a microsyringe and a glass capillary at a rate of 25 nL/min.^16^

### 2.5 Immunostaining

For immunostaining, cultured slices were fixed in 4% PFA at 4°C for 24 hours. The fixed samples were rinsed 3 times with PBS and heated in 10% HistoVT One (06380‐05; Nacalai Tesque) for 20 minutes at 90°C. The slices were then permeabilized and blocked for 1 hour at 4°C in PBS + 0.3% Triton X‐100 with 10% goat serum. The samples were subsequently incubated with primary antibodies in PBS + 0.3% Triton X‐100 with 10% goat serum at 4°C for 48 hours with agitation. The samples were rinsed three times with PBS and then incubated with secondary antibodies in PBS + 0.3% Triton X‐100 with 10% goat serum at 4°C overnight with agitation. The samples were rinsed 3 times with PBS. To label the nuclei, 0.1% Hoechst was added to PBS during the second rinse. After the rinse, the samples were embedded in Permafluor (Thermo Fisher, Waltham, MA, USA). The following primary antibodies were used for immunostaining: mouse anti‐NeuN (1:1000; MAB377; Merck Millipore) and guinea pig anti‐Iba1 (1:500; 324 006, Synaptic System, Goettingen, Land Niedersachsen, Germany). The following secondary antibodies were used for immunostaining: Alexa Fluor 594‐ and 647‐conjugated secondary antibodies (1:500; Thermo Fisher).

### 2.6 Statistical analysis

Data were collected from at least three independent experiments to quantify microglia and neuronal density. Tukey's test after one‐way or two‐way analysis of variance (ANOVA) was used for statistical analysis, and the data were presented as the means ± standard deviation (SD). Data were statistically analyzed by researchers blinded to experimental conditions.

## 3 RESULTS

### 3.1 The effects of KA application in vivo and in slice cultures

**Figure 1:**
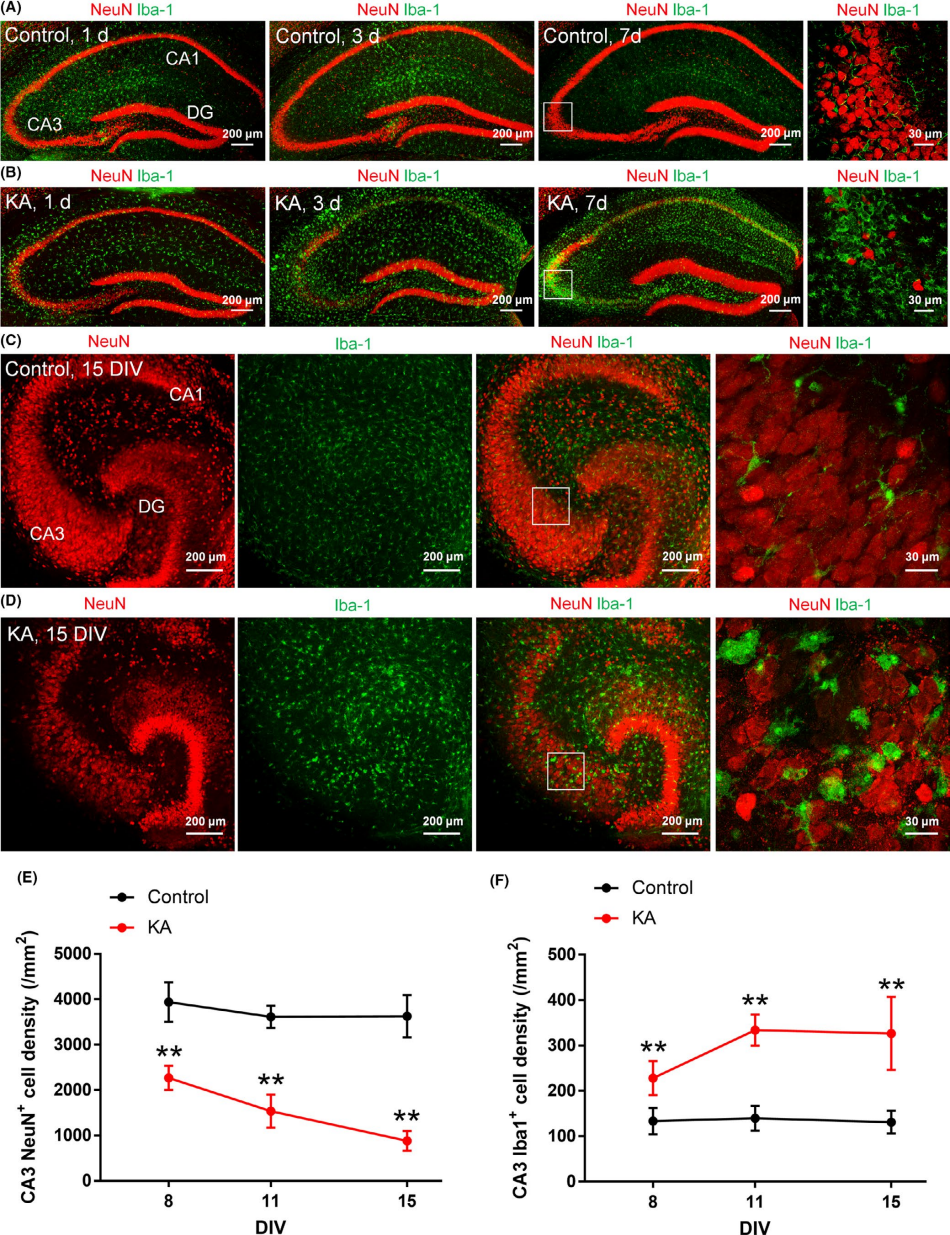
Neuronal death and microglial activation in the hippocampus after kainate (KA) treatment. (A, B) Representative images of the hippocampus of 6‐wk‐old mice immunostained for NeuN and Iba‐1 at 1 d, 3 d, and 7 d after treatment with saline (control, A) or KA (B). Magnified images of the squared areas from 7 d are shown on the right. (C, D) Representative images of control (C) and KA‐treated (D) hippocampal slice cultures immunostained for NeuN and Iba‐1 at 15 d in vitro (DIV). The cultures were treated with KA for 24 h at 7 DIV. Magnified images of the squared areas in the merged images are shown on the right. (E, F) The density of NeuN^+^ cells (E) and Iba‐1^+^ cells (F) in slice cultures at 8, 11, and 15 DIV. **P < .01 vs control; one‐way ANOVA followed by Tukey's test, n = 9‐13 slices. The data represent the mean ± SD

Glutamate receptor overactivation causes the hyperexcitation of neurons, resulting in an excessive influx of calcium and the induction neuronal death. Kainic acid, an agonist of kainite‐class ionotropic glutamate receptors, has been widely used to induce status epilepticus in rodents to model spontaneous recurrent seizures in mTLE.^16^, ^17^ To reproduce the pathological changes of mTLE in mice, we performed intrahippocampal KA injections (20 mmol/L, 50 μL).^16^ and sacrificed the mice 1, 3, and 7 days postinjection to perform immunostaining for the neuronal marker NeuN and the microglial marker Iba1. We confirmed that KA‐induced neuronal loss in the hippocampus, particularly in the CA3 pyramidal cell layer (Figure 1A, 1). Because the remarkable neuronal loss in CA3 accurately reproduces the pathological condition of mTLE.^14^, we mainly focused on changes in CA3. We also found that microglia adopted an ameboid shape, indicating that they were in an activated state (Figure 1A, 1). In addition, we found that microglia accumulated in the pyramidal cell layer where neurons existed 7 days after treatment with KA.

Next, we used hippocampal slice cultures to directly investigate the role of microglia in KA‐induced neuronal death. First, we confirmed whether pathological changes can be reproduced in slice cultures, similar to what occurs in vivo. KA (20 μmol/L) was administered at 7 DIV for 24 hours. We fixed and immunostained cultured slices for NeuN and Iba1 at 8, 11, or 15 DIV (Figure 1C‐F) and found that the neuronal density was significantly decreased (Figure 1E), while the microglial density was significantly increased (Figure 1F) after KA treatment. We also found microglia with an ameboid shape, which suggests that microglia were activated, invaded the neuronal cell layer as observed in vivo (Figure 1D). These results suggest that the pathological changes of neuronal death in mTLE can be reproduced in slice cultures.

### 3.2 The role of microglia in KA‐induced neuronal death in slice cultures

**Figure 2:**
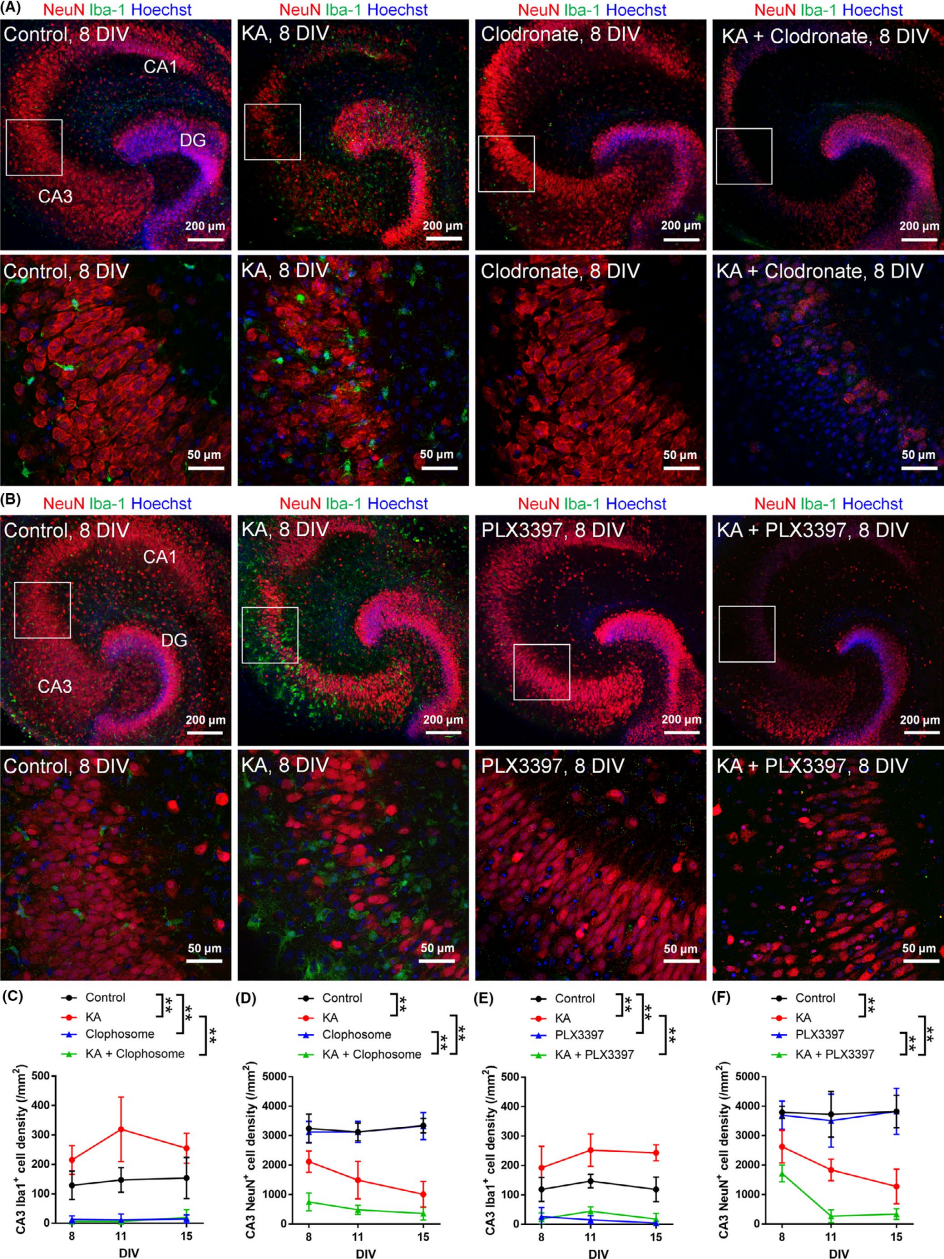
Microglia attenuate KA‐induced neuronal death in slice cultures. (A, B) Representative images of control and KA‐treated hippocampal slice cultures immunostained for NeuN and Iba‐1 at 8 DIV after the removal of microglia by clodronate (A) or PLX3397 (B). The lower images are magnified images of the squared areas in the upper images. (C, D) The density of Iba1^+^ cells (C) and NeuN^+^ cells (D) in the CA3 region of cultured slices treated with KA and clodronate at 8, 11, and 15 DIV. (E, F) The density of Iba1^+^ cells (E) and NeuN^+^ cells (F) in the CA3 region of cultured slices treated with KA and PLX3397 at 8, 11, and 15 DIV. **P < .01; two‐way ANOVA followed by Tukey's test, n = 6‐8 slices. The data represent the mean ± SD

To directly assess the role of microglia in KA‐induced neuronal death, we pharmacologically removed microglia from slice cultures before KA treatment at 7 DIV for 24 hours and histological evaluation of neuronal death was performed by immunostaining (Figure 2A, 2). First, we used clodronate, which inhibits the ATP transporter and has been shown to specifically remove microglia without affecting other cells.^13^, ^14^ Liposomal clodronate (0.5 mg/mL) was administered twice to slice cultures both at 0 and 3 DIV for 24 hours each. We found that microglia were significantly removed in the clodronate‐treated cultures (Figure 2A, 2), whereas the density of neurons was not affected by clodronate (Figure 2A, 2). We also found that clodronate‐induced microglial depletion accelerated and enhanced KA‐induced neuronal death (Figure 2D), suggesting that microglia are neuroprotective.

Second, to confirm that microglia are neuroprotective in KA‐treated slice cultures, we performed another pharmacological depletion of microglia using PLX3397. PLX3397, also known as pexidartinib, depletes microglia by inhibiting the colony‐stimulating factor 1 (CSF1) receptor, the activation of which is required for microglial survival.^15^ PLX3397 was administered from 0 to 7 DIV to deplete microglia, and KA was then administered at 7 DIV. Similar to the effect of clodronate, immunohistochemical analysis revealed that PLX3397 removed microglia and that KA‐induced neuronal loss was enhanced (Figure 2B, 2, 2).

These results together suggest that microglia exert neuroprotective effects against the neurotoxicity of KA. However, the effects of these two drugs on neurons still should be carefully considered. Indeed, we found that the neuronal density was approximately 1 × 10^3^ (cells/ mm^2^) in the clodronate‐treated group and approximately 2 × 10^3^ (cells/ mm^2^) in the PLX3397‐treated group at 8 DIV (Figure 2D, 2). Thus, it is possible that clodronate is more neurotoxic than PLX3397. A previous study reported that clodronate attenuates ATP synthesis in primary cultured neurons.^18^

## 4 DISCUSSION

In this study, we investigated whether microglia are neuroprotective or neurotoxic against KA‐induced neuronal death using pharmacological strategies to deplete microglia in hippocampal slice cultures. We found that the depletion of microglia attenuates KA‐induced neuronal death, which suggests that microglia can be neuroprotective.

We found the robust innervation of microglia by neuronal cell layers as early as 1 day after KA application in slice cultures. Microglia have been shown to quickly respond to changes in neuronal properties, especially when neurons are excessively activated. Previous findings have reported that the number of microglial protrusions increases during stroke induced by KA administration and that microglia extend their protrusions toward neurons.^19^, ^20^ This quick reaction of microglia has been shown to be mediated by ATP released from excited neurons, which acts on microglial P2Y12 receptors. P2Y12 KO mice exhibit a decrease in the number of microglial processes after KA administration and an increased severity of seizures.^19^ In addition, when neurons are excessively activated, microglia structurally wrap axons, inducing the repolarization of neurons and thereby suppressing neurotoxicity resulting from neuronal hyperexcitability.^21^ This indicates that microglia can block excitatory transmission by partially closing axonal channels through physical contact with neurons.

Microglia can also decrease neuronal excitability indirectly. KA treatment increases the release of microglial TNF‐α.^22^, a cytokine known to reduce neuronal excitability through upregulating the neuronal expression of KCNN2 (potassium intermediate/small conductance calcium‐activated channel, subfamily N, member 2).^23^ Consistent with these findings, it has been reported that KA‐induced neuronal cells are increased in mice lacking TNF‐α receptors.^24^ Together, our results support the idea that microglia are potentially neuroprotective against hyperexcitability‐induced neuronal death. However, it should be noted that several studies have reported that microglia can promote neuronal death. The inhibition of microglial activation by minocycline reduces the frequency and severity of neuronal cell death and spontaneous recurrent seizures in a rat lithium‐pilocarpine model of mTLE.^25^ Recent studies have shown that the inhibition of microglial proliferation by blocking CSF1 receptors reduces neuronal cell death.^26^ In our study, both resting and activated microglia were removed from the cultured slices. Since the removal of microglia was performed before the treatment of KA, it is likely that resting microglia were mainly removed. Thus, it is possible that the depletion of resting microglia itself may have caused neuronal hyperexcitability by KA, increasing neuronal death. It should be noted that the timing of microglial removal can affect the results because microglial state, that is, resting or activated, significantly affects their role.^27^ As the role of microglia can change depending on the process of diseases.^9^, it is important to carefully investigate the transitory state of the microglial role in neuroprotection and neurotoxicity.

In summary, we found that the removal of microglia promotes KA‐induced neuronal cell death in the hippocampal slice cultures. These results support previous findings that microglia are neuroprotective against hyperexcitability‐induced neuronal cell death. Though we have not examined the molecular mechanisms underlying microglia‐dependent neuroprotection, our in vitro model will be useful to pharmacologically and genetically assess the molecular mechanisms in future studies.

## CONFLICT OF INTEREST

Authors declare no conflict of interest.

## AUTHOR CONTRIBUTIONS

TA conducted the experiments, analyzed the experimental data, and wrote the manuscript. RK designed and planned the project and wrote the manuscript. YI discussed the results and commented on the manuscript.

## ETHICAL APPROVAL

All experiments were performed with the approval of the animal experiment ethics committee at the University of Tokyo and according to the University of Tokyo's guidelines for the care and use of laboratory animals.

## Data Availability

We have made our data publicly available in Supporting Information.
